# Revealing the effects of amino acid, organic acid, and phytohormones on the germination of tomato seeds under salinity stress

**DOI:** 10.1515/biol-2022-0892

**Published:** 2024-06-11

**Authors:** Faika Yarali Karakan, Haluk Caglar Kaymak, Selen Akan, Sezai Ercisli, Amine Assouguem, Riaz Ullah, Essam A. Ali, Hafize Fidan

**Affiliations:** Department of Horticulture, Faculty of Agriculture, Kilis 7 Aralik University, Kilis, Turkey; Faculty of Agriculture, Department of Horticulture, Atatürk University, Erzurum, Turkey; Department of Horticulture, Faculty of Agriculture, Ankara University, Ankara, Turkey; Department of Plant Protection and Environment, École Nationale d’Agriculture de Meknès, Km.10, Route Haj Kaddour, B.P.S/40, Meknes, 50001, Morocco; Laboratory of Functional Ecology and Environment, Faculty of Sciences and Technology, Sidi Mo-hamed Ben Abdellah University, Imouzzer Street, P.O. Box 2202, Fez, 30000, Morocco; Department of Pharmacognosy, College of Pharmacy, King Saud University, Riyadh, Saudi Arabia; Department of Pharmaceutical Chemistry, College of Pharmacy, King Saud University, Riyadh, 11451, Saudi Arabia; Department of Tourism and Culinary Management, Faculty of Economics, University of Food Technologies, Plovdiv, Bulgaria

**Keywords:** abiotic stress, biochemical pathways, seed physiology, *Solanum lycopersicum*

## Abstract

Salinity accumulation poses a threat to the production and productivity of economically important crops such as tomatoes (*Solanum lycopersicum* L.). Currently, salt tolerance breeding programs have been limited by insufficient genetic and physiological knowledge of tolerance-related traits and a lack of an efficient selection domain. For that purpose, we aimed to determine the ability of tomato cultivars to tolerate salt based on seed traits by multiple biochemical pathways. First, we tested three tomato cultivars according to their response to different sodium chloride (NaCl) concentrations (0, 6.3, 9.8, 13.0, and 15.8 dS m^−1^) and then we analysed their amino acids, organic acids, and phytohormones. Considering the results of germination traits, it is possible to conclude that cultivar H-2274 was more tolerant to salt stress than others. As a result, multivariate discriminant analysis including principal component analysis and two-way hierarchical clustering analyses were constructed and demonstrated that tomato cultivars were separated from each other by the amino acid, organic acid, and phytohormone contents. Considering germination traits of tomato seeds, cv. ‘H-2274’ was more tolerant to salinity than others depending on high proline (29 pmol µl^−1^) and citric acid (568 ng µl^−1^) assays. Biochemical variability offers a valuable tool for investigating salt tolerance mechanisms in tomatoes, and it will be appreciated to find high-tolerant tomato cultivar(s) to saline conditions. Also, the findings of this study have significant potential for practical applications in agriculture, particularly in developing salt-tolerant tomato cultivars to enhance productivity in saline environments and address socio-economic challenges.

## 1 Introduction

Tomato (*Solanum lycopersicum* L.) is one of the most widely grown vegetables [1]. Tomatoes are widely consumed for their nutrition and health effects [2]. However, a variety of biotic or abiotic stress factors have a negative impact on tomato growth and development [3]. Among abiotic factors, salt stress is the major environmental stress, affecting about 23% of cultivated lands worldwide, especially in arid and semi-arid regions, and decreasing one-third of crop production [4,5]. Therefore, dealing with salinity is crucial in achieving sustainable agricultural practices and meeting current and future food demand globally. Many commercial tomato cultivars range from moderately sensitive to sensitive to salinity. Hereby, in the future decades, it is critical to choose or produce salinity-resistant cultivars.

Salinity stress, with sodium chloride (NaCl) being the predominant salt, has had a detrimental impact on tomato seed germination and early seedling growth, which are the most decisive stages in species establishment. In particular, salinity inhibits water uptake of plants by causing ionic toxicity and osmotic stress as well as reducing germination rates [3,5]. In addition, salinity affects other aspects of plant growth, such as biochemical metabolism. Hence, plants respond to salt through diverse strategies and approaches that modulate self-physiological and biochemical mechanisms, providing new insights into how plants adapt to saline conditions. Plants alter biochemical functions such as nitrogenous compounds, amino acids, and organic acids to help alleviate the osmotic stress induced by salinity [6,7,8].

Amino acids tend to accumulate in the tissues of plants when exposed to salt stress. For instance, proline enhances antioxidant machinery, maintains protein structures, and stabilizes cell membranes during salt stress [5]. Thus, stress proteins such as proline could be used as a selective parameter for salt resistance [7]. Organic acid metabolism is of primary importance at the cellular level for various biochemical pathways such as amino acid biosynthesis, energy production, and modifying plant adaptability to the environmental condition. When plants are subjected to salt stress, they synthesize short-chain organic acids to maintain cell turgor, allowing them to absorb excess sodium while producing organic acids [3,9]. Gong et al. [10] reported that NaCl increased oxalic, tartaric, and malic acids in tomato roots. In addition, determining the amount of amino acids and organic acids in seeds and revealing their effects on germination under salt stress may aid in the identification of cultivars or genotypes resistant to salt stress at the first selection stage of breeding studies. Similar to amino acids and organic acids, phytohormones play an important role in controlling plant responses to abiotic stress, allowing the plant to focus its own resources on stress resistance [11]. Plants upregulate the levels of endogenous phytohormones, such as abscisic acid (ABA), salicylic acid (SA), and ethylene under stress conditions [5,12]. In fact, ABA and ethylene are stress hormones with specific roles in regulating salt stress tolerance and resistance [6].

A number of studies have been conducted to investigate the effects of organic acids, amino acids, and phytohormones on seed germination and the growth of tomato plants or roots under salt stress. However, to the best of our knowledge, no studies have been conducted on the involvement of tomato seed biochemical components in germination parameters under salt stress. Focusing on this idea, to fill this gap, we aimed to determine the role of some biochemical components such as phytohormones, amino acids, and organic acids in tomato seed salinity resistance and also to investigate whether the biochemical content of seeds could be used to predict salinity resistance.

## 2 Materials and methods

### 2.1 Plant material

The seeds of three tomato cultivars (*S. lycopersicum* L. ‘Falcon,’ ‘Rio Grande,’ and cultivar H-2274) were selected in this study because of their strong plant structure [13]. These cultivars were obtained from various commercial seed companies in 2021.

### 2.2 Salt stress treatments

The seeds were sterilized with 5% sodium hypochlorite solution for 5 min, and then they were washed three times with distilled water [14]. A standard germination test was conducted using four replications of 50 seeds from each cultivar in petri dishes (with 9 cm diameter) and spread across two filter paper sheets moistened with 0 (control), 6.3, 9.8, 13.0, and 15.8 dS m^−1^ NaCl solutions. They were kept at 20°C in a growth chamber with a photoperiod of 9/15 h each day [15]. The petri dishes were placed in a complete randomized design with four replications. Visible-radicle protrusion (≥2 mm) was the criterion of germination. Germinated seeds were counted at 24-h intervals for 14 days. At the end of the standard germination test, the germination percentage in the first count (5th day), germination percentage (%), germination speed (%), and radicle length (cm) were determined. The germination percentage was calculated by dividing the number of germinated seeds in any Petri dishes by the total number of seeds, then multiplied by 100 [16,17]. Germination speed was calculated according to the equation (germination speed = germination percentage in 1st day/1 + … + germination percentage 10th day/10’) of Kaymak [18]. To assess the vigour, seeds were sown in plastic trays (20.5 × 11.0 × 6.2 cm) filled with sphagnum peat [19,20]. The plastic trays were placed in laboratory conditions for a period of 3 weeks. The trays were irrigated daily with five (0, 6.3, 9.8, 13.0, and 15.8 dS m^−1^) different NaCl solutions, which were derived from distilled water by the use of NaCl. In addition, small holes were drilled so that excess water can drain naturally from the bottom of the boxes. Counts started as seedlings began to emerge and continued until the end of the 3 weeks. Seedlings were considered to have emerged when their cotyledons were free of the soil surface.

### 2.3 Biochemical assessments

#### 2.3.1 Amino acids

Amino acid content was analysed as described by Gunes et al. [21]. For extraction, 1 g seed samples were taken and placed into a tube; then, 0.1 N HCl solution was added to it, and then prepared samples homogenized with a homogenizer (IKA-Labortechnik, Ultra-turrax T25) for 1.5 min at 9,500 × *g*. The homogenate was incubated at 4°C for 12 h. Subsequently, the mixture was centrifuged for 50 min at 1,200 × *g*. The supernatant was separated and filtered, followed by a 0.22 μm membrane filter (Millipore, USA). The prepared filtrates were transferred to glass vials and used for high-pressure liquid chromatography (HPLC) analysis. The amino acid derivatives were determined using HPLC (Agilent-1200) equipped with a Zorbax Eclipse-AAA column (4.6 × 150 mm, 3.5 µm) and a UV visible detector. A (40 mM NaH_2_PO_4_ [pH 7.8]) and B (acetonitrile/methanol/water [45/45/10, v/v/v]) solutions were used gradient at a flow rate of 2 ml min^−1^ as mobile phase. Column temperature was set as 40°C, and readings were done at 254 nm. The amino acids were identified through comparison to standards. *O*-phthaldialdehyde, fluorenylmethyl-chloroformate, and 0.4 N borate were used for derivation processes in an autosampler. Arginine, histidine, isoleucine, leucine, lysine, methionine, phenylalanine, proline, threonine, and valine quantities from seed samples were determined as pmol µl^−1^ after a 26 min derivation process in HPLC.

#### 2.3.2 Organic acids

To determine the organic acid content of tomato seed, a 1 g seed sample was homogenized with 10 ml of doubly distilled water using an IKA-Labortechnik, Ultra-turrax T25 homogenizer at 24,000 rpm for 20 s. After centrifugation at 1,200 rpm for 50 min, the supernatant was filtered with a 0.22 µm membrane filter, collected in glass vials, and then analysed with an Agilent-1200 HPLC equipped with a Zorbax Eclipse-AAA column (4.6 × 250 mm, 5 µm) and UV visible detector. The flow rate was 1 ml min^−1^, the column temperature was 25°C, and readings were taken at 220 nm. The results were calculated based on external citric acid, fumaric acid, malic acid, succinic acid, oxalic acid, and tartaric acid standards using 25 mM potassium phosphate (pH 2.5) as the mobile phase. Data were expressed as ng µl^−1^.

#### 2.3.3 Phytohormones

The extraction and quantification were carried out following the method described by Gunes et al. [22] with partial modifications. The tomato tissue was ground in liquid nitrogen, and 80% methanol, which was held at −40°C, was added. Then, the mixture was homogenized with a homogenizer (Ultra-Turrax) for 10 min and then incubated in dark conditions for 24 h. Afterwards the solution was first filtered with filter paper and second filtered through a 0.45 m pore filter. The obtained samples were dried at 35°C using evaporator pumps. Subsequently, the dried samples were dissolved in 0.1 M KH_2_PO_4_ (pH 8.0). To separate fatty acids, the extracts were centrifuged at 5,000 × *g* at 4°C for 1 h. Then, 1 g of polyvinyl polypyrrolidone (PVPP) was added to the supernatant in order to separate phenolic and colour matters (Gunes et al., [22]). To separate PVPP from the supernatant, the supernatant was filtered through Whatman No. 1 filter paper, and the Sep-Pak C-18 (Waters) cartridge was used for further specific separation. After adsorption of phytohormones by cartridge, the solution was transferred to vials and directly injected into HPLC (Agilent 1200) equipped with C-18 column Zorbax Eclipse (4.6 × 150 mm, 3.5 µm), and a UV detector was used for separation and determinations. The mobile phase consisted of acetonitrile and water (13:87, v/v) at pH 4.98 with a flow rate of 1.2 ml min^−1^, and the column temperature was 25°C. The readings were done at 265 nm, gibberellic acid (GA_3_), SA, indole acetic acid (IAA), ABA, ethylene, and zeatin were determined and given as ng µl^−1^.

### 2.4 Statistical analysis

This experiment was designed as a randomized complete design, with each analysis replicated four times. All statistical analyses were performed using JMP pro version 14 (SAS Institute, NC, USA). Significant differences between NaCl treatments were evaluated by one-way analysis of variance (ANOVA). When significant effects were detected, the Tukey test was performed to compare the NaCl treatments within each cultivar at a significance level of 0.05. Principal component analysis (PCA) and hierarchical cluster analysis (HCA) were used to describe patterns of variation in the biochemical profile of cultivars.

## 3 Results and discussion

### 3.1 Effects of NaCl treatments on germination parameters of three tomato cultivars

**Figure 1:**
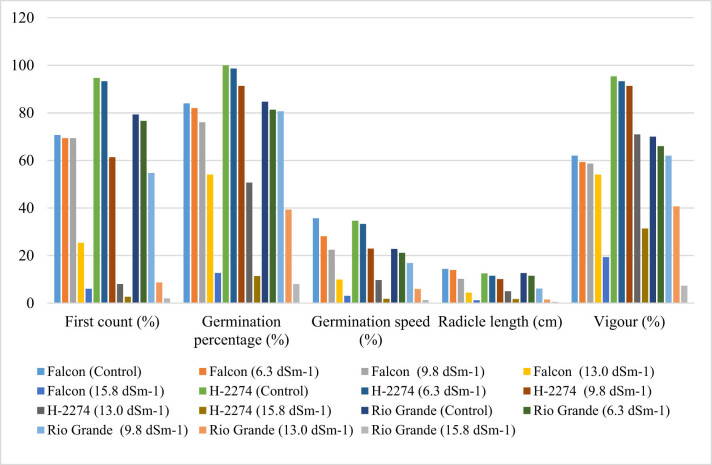
First count, germination percentage and speed, radicle length, and vigour in tomato cultivars under different NaCl treatments.

In the experiment, different concentrations of NaCl showed a significant effect on the germination percentage in the first count, germination percentage and speed, vigour, and radicle length of the different tomato cultivars (Table 1 and Figure 1).

**Table 1:** First count, germination percentage and speed, radicle length, and vigour in tomato cultivars under different NaCl treatments

	NaCl (dS m^−1^)	Falcon	H-2274	Rio Grande
First count (%)	Control	70.66 ± 3.05bc*	94.66 ± 1.15a	79.33 ± 4.61b
	6.3	69.33 ± 5.77bc	93.33 ± 5.03a	76.66 ± 1.15b
	9.8	69.33 ± 5.77bc	61.33 ± 3.05cd	54.66 ± 2.30d
	13.0	25.33 ± 8.32e	8.00 ± 4.00f	8.66 ± 2.30f
	15.8	6.00 ± 5.29f	2.66 ± 1.15f	2.00 ± 0.00f
Germination percentage (%)	Control	84.00 ± 3.46ab	100.00 ± 0.00a	84.66 ± 2.30ab
	6.3	82.00 ± 2.00b	98.66 ± 2.30a	81.33 ± 1.15b
	9.8	76.00 ± 4.00b	91.33 ± 4.16ab	80.66 ± 4.16b
	13.0	54.00 ± 12.16c	50.66 ± 1.15c	39.33 ± 4.16c
	15.8	12.66 ± 10.26d	11.33 ± 7.57d	8.00 ± 5.29d
Germination speed (%)	Control	35.67 ± 3.21a	34.63 ± 1.90a	22.76 ± 2.43cd
	6.3	28.14 ± 2.68bc	33.28 ± 4.24ab	21.15 ± 0.52d
	9.8	22.41 ± 1.51cd	22.92 ± 1.14cd	16.87 ± 1.31d
	13.0	9.90 ± 2.22e	9.68 ± 0.54e	5.92 ± 0.55ef
	15.8	3.07 ± 2.60f	1.80 ± 1.24f	1.26 ± 0.67f
Radicle length (cm)	Control	14.38 ± 0.89a	12.50 ± 0.00ab	12.61 ± 1.56ab
	6.3	13.93 ± 1.11a	11.53 ± 1.07ab	11.51 ± 2.12ab
	9.8	10.19 ± 1.58b	10.08 ± 0.83b	6.06 ± 1.46c
	13.0	4.44 ± 0.78cd	4.96 ± 0.57c	1.49 ± 0.86de
	15.8	1.21 ± 0.41de	1.69 ± 0.32de	0.46 ± 0.10e
Vigour (%)	Control	62.00 ± 0.00bcd	95.33 ± 1.15a	70.00 ± 3.46b
	6.3	59.33 ± 1.15cd	93.33 ± 3.05a	66.00 ± 0.00bc
	9.8	58.67 ± 2.31cd	91.33 ± 2.31a	62.00 ± 0.00bcd
	13.0	54.00 ± 0.00d	71.00 ± 6.08b	40.67 ± 5.77e
	15.8	19.33 ± 8.08f	31.33 ± 2.31e	7.33 ± 2.31g

*: Values within rows followed by different letters differ significantly by Tukey’s multiple range tests at 5%.

The first count of seeds ranged from 2.00 to 94.76%, with the germination percentage ranging from 8.00 to 100% among the cultivars. For each cultivar, as the salt concentration increased, the germination percentage and speed, vigour, and radicle length decreased. It has been reported that the most harmful effects of saline conditions are seen in the germination phase by inhibiting germination significantly [23,24]. As expected, the highest germination percentage and speed, vigour, and radicle length were obtained from the control, which was non-treated with salt. These results are in conformity with those reported by Sardoei and Mohammadi [14], Kumar et al. [23], and Seth and Kendurkar [25]. The germination percentage and speed, vigour, and radicle length of tomato cultivars in the control were 100.00, 35.67, 95.33, and 14.38% on average, respectively. Similarly, Kumar et al. [23] stated that the germination percentage was lowest (34.31%) in the cross EC-620428 × EC-620557, genotype NS-516 exhibited 87.93%, 85.66%, 85.54%, 82.87%, and EC-620428 × EC-520078 exhibited minimum 32.82, 26.24, 26.24, and 19.82% of germination percentage values at 50, 75, and 100 mM concentration of NaCl, respectively. When taking into account the NaCl concentrations, the highest germination percentage (54.00%) was determined in the cv. ‘H-2274,’ which tolerated salinity up to higher NaCl concentration (13.0 dS m^−1^), followed by the cv. ‘Falcon’ (50.66%), and the cv. ‘Rio Grande’ (39.33%). Considering the results of germination percentage, it could be concluded that the cv. ‘H-2274’ was more tolerant to salt stress than others based on the reduction in germination percentage at the highest NaCl concentration (15.8 dS m^−1^). It is worthy to mention that the cv. ‘Rio Grande’ represented more sensitivity by reducing drastically (90.56%) to saline conditions, especially in the highest concentrations (15.8 dS m^−1^). Consistent with our results, previous reports have stated that the salt tolerance of tomato cultivars varies depending on species manifested by physiological adaptations of the plants [1,6].

Apart from the harmful effects of salinity on germination percentage, salinity prolongs the complete germination elapsed time [26]. In this study, the germination speed of the studied tomato cultivars was affected by NaCl concentrations presented in Table 1. The obtained germination percentage results revealed that the cultivars ‘Falcon,’ ‘H-2274,’ and ‘Rio Grande’ exhibited reduction up to 72.00%, 72.04%, and 73.99%, respectively. A similar observation reported by Singh et al. [27] showed that 3% NaCl-treated tomato seeds required 100% more time to germinate than those treated with 1% NaCl. Adilu and Gebre [28] also found that the germination speed of tomato seeds was significantly affected by the combined effects of variety and salinity, and 4 dS m^−1^ NaCl treatment had an adverse effect on the germination speed of tomato cultivars.

Regarding vigour, data presented in Table 1 demonstrated that with each cultivar, increasing NaCl concentration led to progressively decreased vigour. Our results are in conformity with previous studies in tomato lines [29]. It is obvious that salinity stress caused a significant decrease in vigour, and a considerable decrease was recorded in the cv. ‘Rio Grande’ (90%) among cultivars at the highest NaCl concentration (15.8 dS m^−1^). While the cv. ‘Rio Grande’ was susceptible to salinity levels, and the cultivar ‘H-2274’ had higher tolerance to salinity compared to the other cultivar.

With respect to the radicle length, salinity significantly decreased the seedling growth, leading to a reduction in radicle length. Similar results were also observed in tomato and spinach [30]. At 15.8 dS m^−1^, the radicle length of the seedlings was reduced in cv. ‘Rio Grande’ (96.35%), and in the cv. ‘Falcon’ (91.58%) preceded by cv. ‘H-2274’ (86.48%). This is consistent with the findings by Seth and Kendurkar [25], which showed that salinity leads to a 77.64% reduction in root length at a higher level (100 mM NaCl).

### 3.2 Relationship between amino acids in tomato seeds and salt tolerance

**Figure 2:**
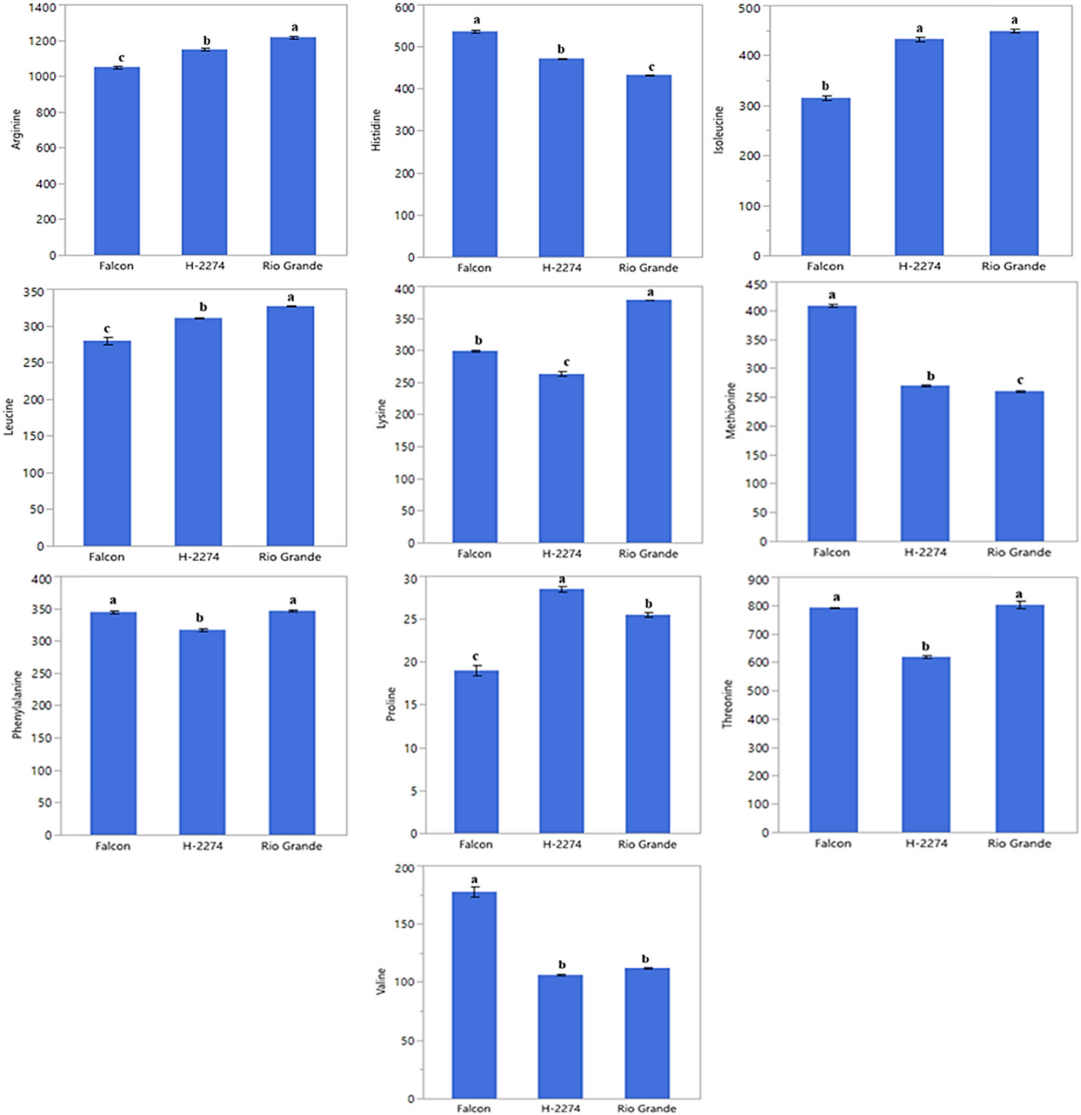
Detected amino acids in tomato cultivars (pmol µl^−1^) (the error bars represent confidence intervals; different letters indicate the significant results are those for which the *p*-value was lower than 0.05).

**Figure 3:**
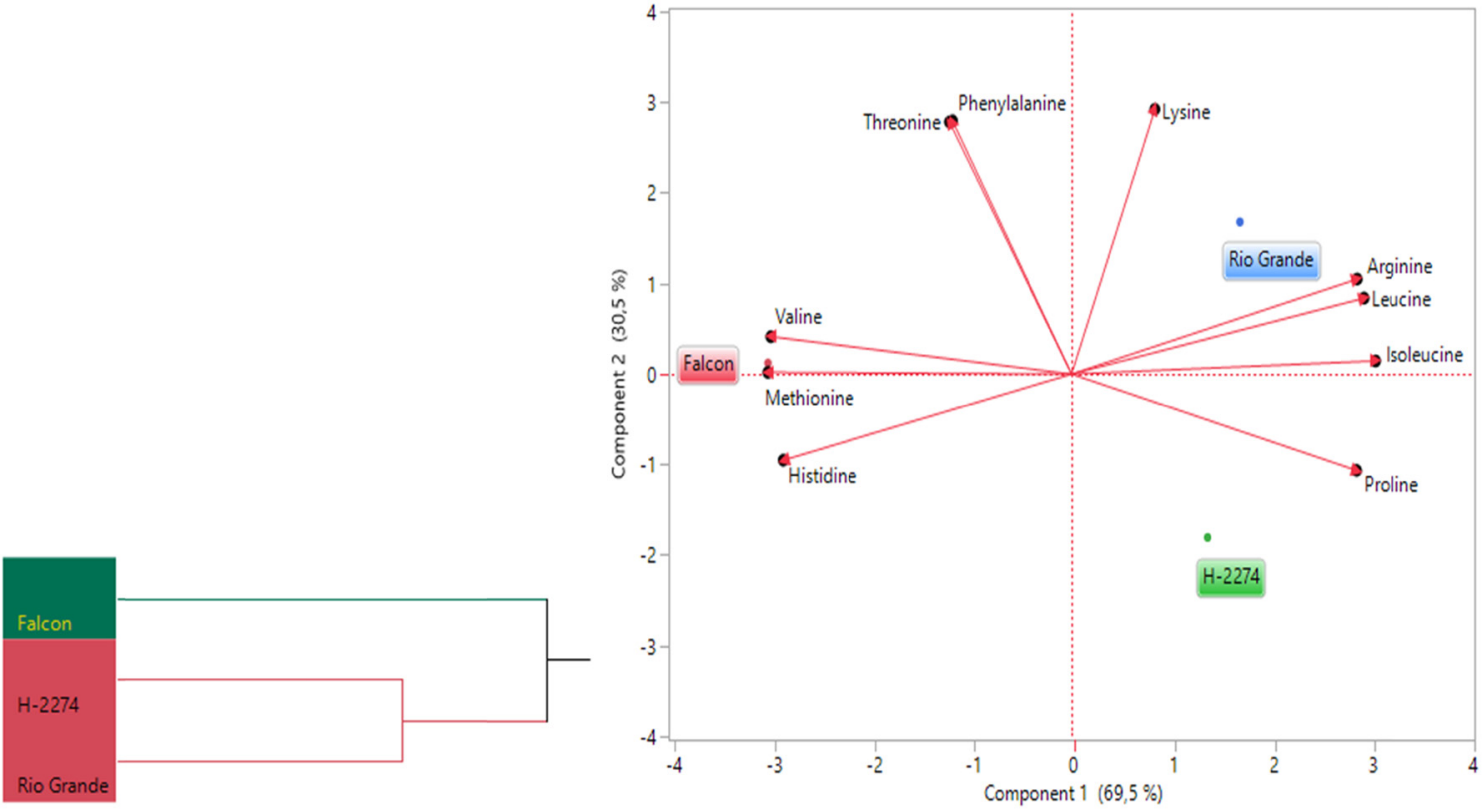
Dendogram obtained by two-way hierarchical clustering analysis (HCA) shows the distribution of tomato varieties according to amino acid content (left). The biplot plot graph obtained by principal component analysis shows the relationships between tomato cultivars and amino acids (right).

The amino acid composition of three tomato cultivars was determined as depicted in Figures 1 and 2. A total of 10 amino acids including arginine, histidine, isoleucine, leucine, lysine, methionine, phenylalanine, proline, threonine, and valine have been identified in tomato cultivars (Figure 2). There were significant differences in amino acid concentration among cultivars. Arginine, threonine, and isoleucine were found in high amounts in tomato cultivars, accounting for more than 50.84, 55.46, and 56.71% of the total studied amino acid contents in cultivars ‘Falcon,’ ‘H-2274,’ and ‘Rio Grande,’ respectively. Other amino acids, histidine, leucine, lysine, methionine, phenylalanine, proline, and valine, were found in small amounts. The dendrogram and biplot graph also created and revealed that there was a clear separation among the cultivars (Figure 3). Figure 3 shows cluster I comprised cv. ‘Falcon’ was grouped individually, and the other cluster (cluster II) consisted of two cultivars (‘H-2274’ and ‘Rio Grande’), which were divided into two subgroups. In the case of PCA, it was calculated by using the scatter biplot method that PC1 (69.5%) and PC2 (30.5%) contents were constituted to be 100% of the total variation. In addition, the relationships between cultivars and amino acid content were computed by principal components biplot analysis and given in Figure 3.

When the relationships among cultivars and amino acids are interpreted by means of a biplot graph, it can be clearly correlated relationship between them. In terms of PCA results, arginine, leucine, isoleucine, and proline were positively related to each other. Likewise, valine, methionine, and histidine were positively related to each other. However, these two groups demonstrated that the correlation relationship with these properties is negative and has a low degree of overlap. Conversely, the cultivar ‘Falcon’ presents higher values in histidine, methionine, and valine than the rest of the cultivar. In addition, cv. ‘Rio Grande’ is found to be rich in arginine, leucine, and lysine; conversely, the cv. ‘H-2274’ has a high value only in proline (Figures 3 and 8).

According to experience, to find any cultivar of species tolerant to saline conditions, the results of amino acid assays of these cultivars can be considered. Hence, as aforementioned in the germination experiments, the cultivar H-2274 was salt tolerant, the cv. ‘Falcon’ and cv. ‘Rio Grande’ were found as sensitive. Depending on the high proline content in the cv. ‘H-2274’ seeds, it can be an inference that it has a high tolerance to salinity. In this respect, previous studies have stated that proline is an important amino acid known to accumulate in plants when exposed to salinity, and it has been used as a tool to measure the saline stress [31]. Similar conclusions were drawn in tomato cultivars by Torre-González et al. [8] and Shin et al. [32], indicating that the amount of proline in the stem and leaf of tomato cultivars increased under saline conditions. Similarly, Abouelsaad et al. [33] reported that the level of proline in leaf tissues of tomatoes was exposed to NaCl increased by 4.71 fold. Similar correlations have been described by Gharsallah et al. [5], who reported an increase of proline concentration in the leaves of the tomato genotype ‘San Miguel,’ which is known to have high salt tolerance with increasing salt stress. Our results corroborate these findings related to the high salt-tolerant cultivars (cv. ‘Rio Grande’ and cv. ‘H-2274’), which have high arginine and proline content. As outlined by Zushi and Matsuzoeba [9], proline, phenylalanine, histidine, threonine, and arginine were higher in the cultivar ‘House Momotaro’ treated with the 100 mM salt, whereas proline and phenylalanine were higher in the cultivar ‘Mini Carol’ treated at the same concentration. Zhang et al. [3], in experiments involving phenylalanine, glutathione, cysteine, methionine, arginine, proline, alanine, aspartate, glutamate, glycine, serine, and threonine role in tomato, reported significantly enriched in the adaption to salt stress. According to these authors, arginine is a vital amino acid to occupy a precursor to the synthesis of other amino acids, and proline plays an important role during the salt stress response.

### 3.3 Relationship between organic acids in tomato seeds and salt tolerance

**Figure 4:**
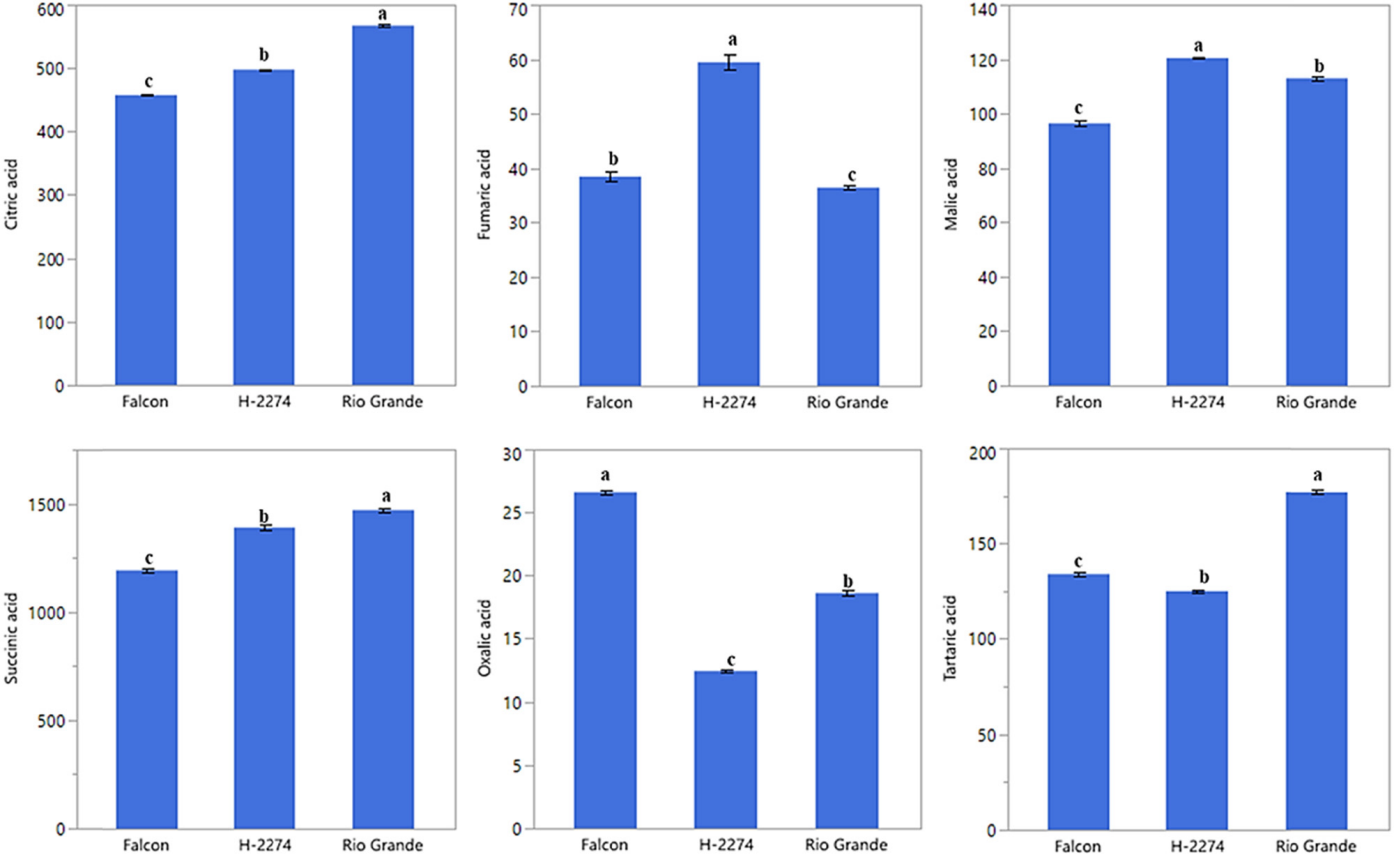
Detected organic acids in tomato cultivar seeds (ng µl^−1^) (the error bars represent confidence intervals; different letters indicate the significant results are those for which the *p*-value was lower than 0.05).

**Figure 5:**
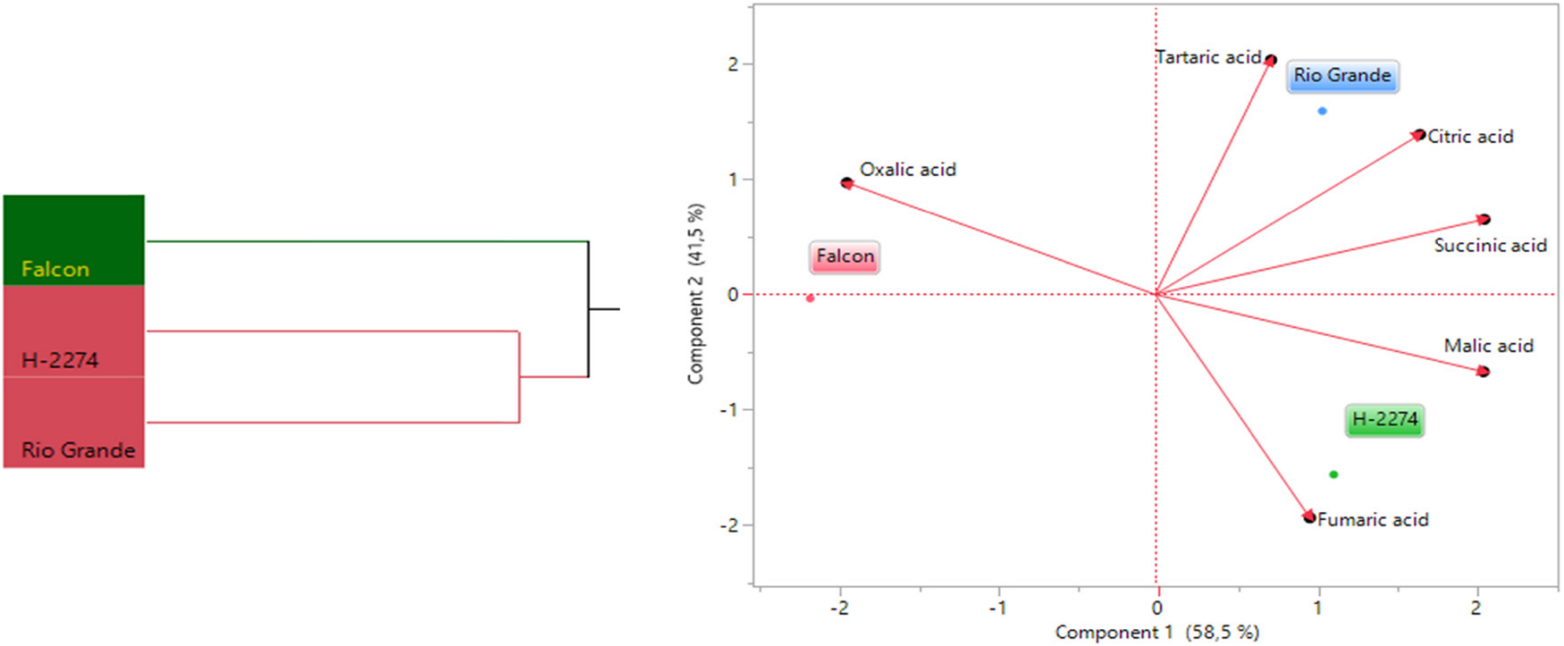
Dendogram obtained by two-way HCA shows the distribution of tomato varieties according to organic acid content (left). The biplot plot graph obtained by principal component analysis shows the relationships between tomato cultivars and organic acids (right).

The analyses of organic acid content from seeds of three tomato cultivars identified six organic acids consisting of citric acid, fumaric acid, malic acid, succinic acid, oxalic acid, and tartaric acid by HPLC (Figure 4). We next determined similarities and dissimilarities between cultivars and organic acids, as graphed in Figure 5. The organic acids assay revealed that it ranged from 1,192 to 1,470 ng µl^−1^, from 458 to 568 ng µl^−1^, from 125 to 177 ng µl^−1^ with succinic, citric, and tartaric acid being the major constituents in tomato seeds. As for cultivars, the succinic acid values were 1,470, 1,389, and 1,192 ng µl^−1^ in the cv. ‘Rio Grande,’ ‘H-2274,’ and ‘Falcon,’ respectively. Citric acid (568 ng µl^−1^), succinic acid (1,470 ng µl^−1^), and tartaric acid (177 ng µl^−1^) were most widely detected in the ‘H-2274’ cultivar. The content of malic acid and fumaric acids was significantly higher in the ‘Falcon’ than in other cultivars. The cultivar ‘Rio Grande’ showed the highest oxalic acid content (Figures 5 and 8). The dendrogram and biplot graph presented two major clusters, and each cluster showed the existence of diversity and similarity in the cultivars based on the organic acids. As seen in Figure 5, cluster I comprised the ‘Falcon’ and the other cluster (cluster II) consisted of two cultivars (‘H-2274’ and ‘Rio Grande’), which were divided into two subgroups. In addition, the relationships between the cultivars and organic acid contents are given in Figure 5. According to the relations, a positive correlation was found among succinic acid, citric acid, and tartaric acid. Also, malic acid and fumaric acid were positively correlated with each other. It was no coincidence; Chen et al. [34] explored the mutual conversion process between fumaric acid and malic acid under different conditions. Additionally, malic acid, fumaric acid, and oxalic acid showed a negative and low correlation.

Tolerance to salinity is a composite phenomenon involving a lot of metabolic pathway modulation [35]. Our study involving organic acid content in tomato seeds contributed important evidence in understanding the role of their tolerance to salt stress. A very common phenomenon of organic acids including oxalic acid, tartaric acid, malic acid, citric acid, and succinic acid is an accumulation of these, an inevitable consequence of salt stress in tomatoes, as mentioned in previous reports [10]. Comparing organic acid content, Zushi and Matsuzoe [9] determined the higher citric acid content in the salt-stressed fruit, but the malic acid content remained unchanged. Therefore, it could be said that the high amount of citric acid in the seed provides an advantage in salt response. When taking into account of aforesaid approaches, from the present study, it was noted that the cultivar ‘H-2274,’ having higher citric acid and malic acid had high salt tolerance.

### 3.4 Relationship between phytohormones in tomato seeds and salt tolerance

**Figure 6:**
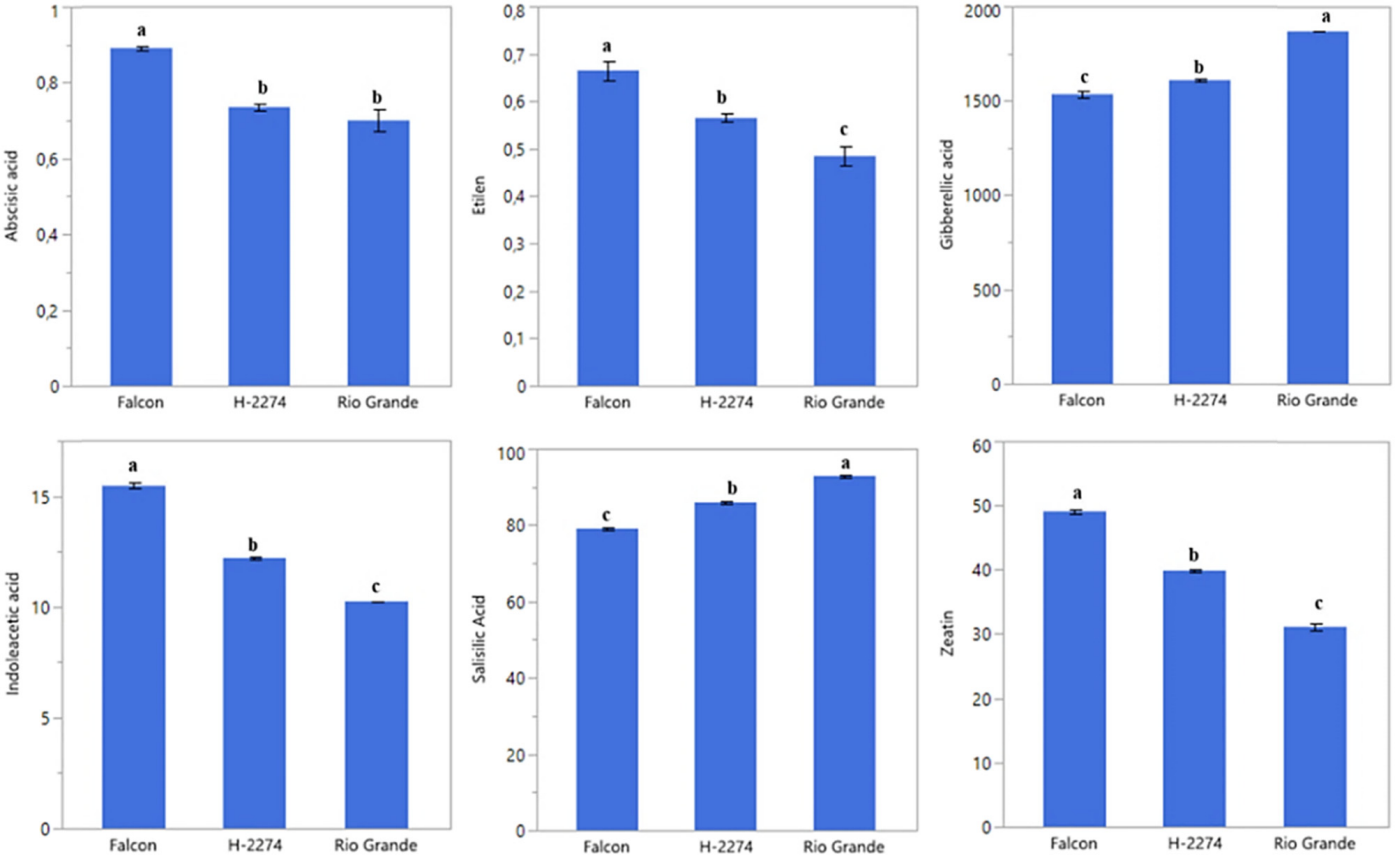
Detected phytohormones in the seed of tomato cultivars (ng µl^−1^) (the error bars represent confidence intervals; different letters indicate the significant results are those for which the *p*-value was lower than 0.05).

**Figure 7:**
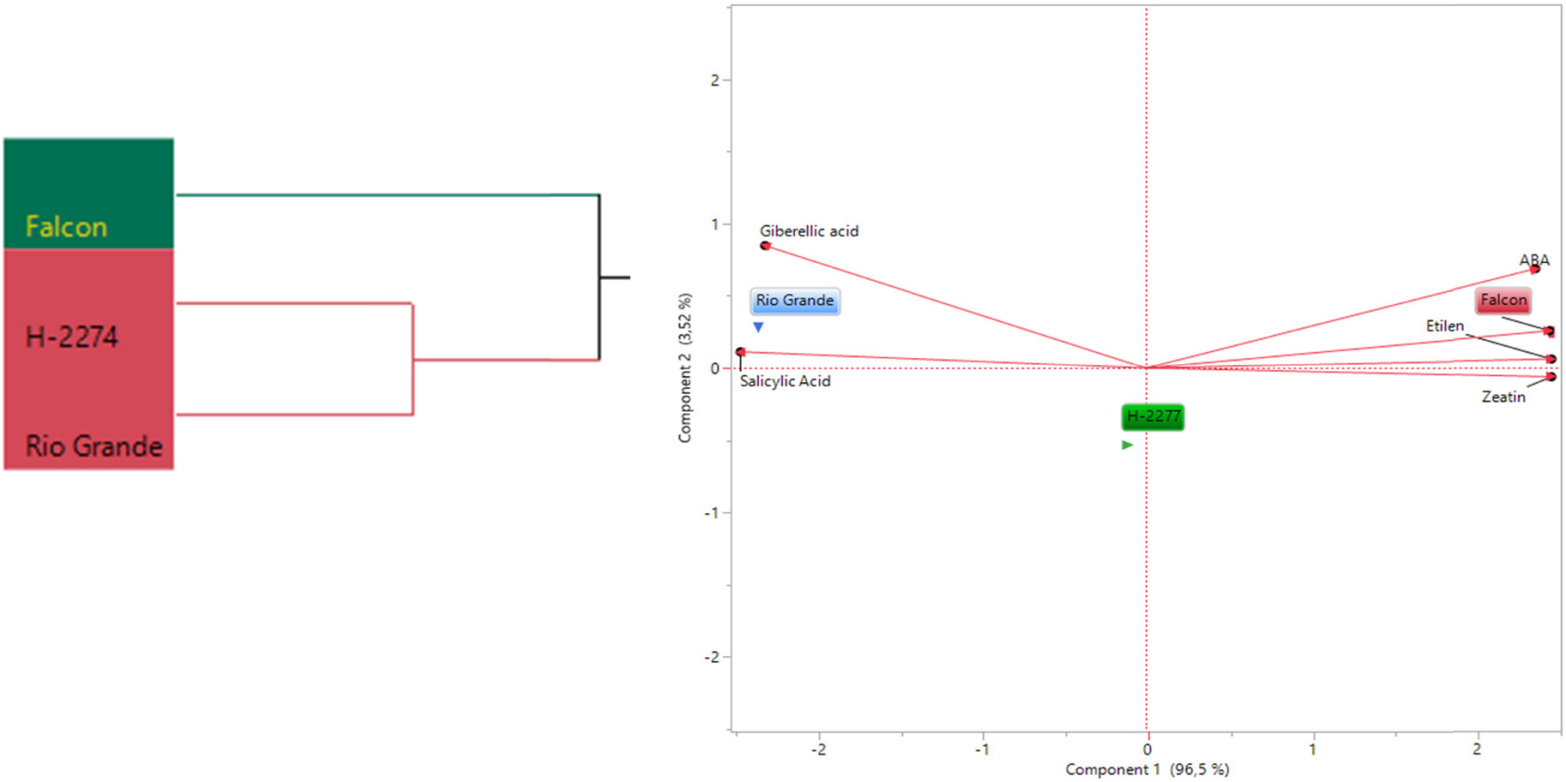
The dendogram obtained by two-way (HCA shows the distribution of tomato varieties according to phytohormones content (left). The biplot plot graph obtained by principal component analysis shows the relationships between tomato cultivars and phytohormones content (right).

**Figure 8:**
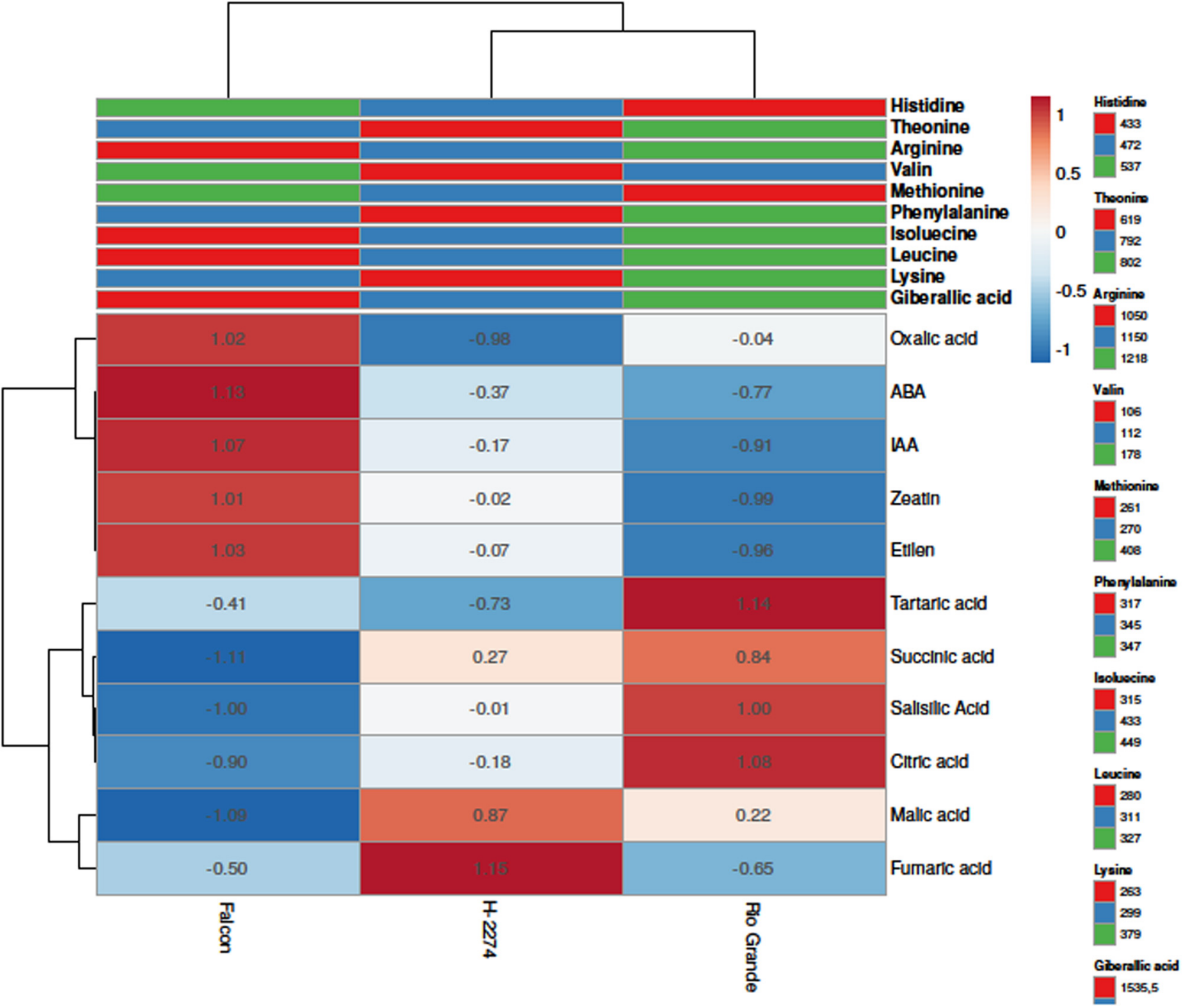
The heat map of the principal component analysis was conducted to demonstrate the similarities and dissimilarities among the biochemical composition of the seed of three tomato cultivars in terms of the content of common detected amino acids, organic acids, and phytohormones (in the heat map, rows and columns are grouped by correlation distance and average linkage).

A total of six phytohormones consisting of ABA, ethylene, GA_3_, IAA, SA, and zeatin were identified in the seed of three tomato cultivars (Figure 6). Like amino acid and organic acid contents, these phytohormones are differentially altered in tomato seeds. GA_3_, SA, and zeatin were present in high quantities. The values of GA_3_, SA, and zeatin were 1,535, 1,609, and 1,870 ng µl^−1^; 79.15, 85.94, 85.94, and 92.89 ng µl^−1^; 49.07, 39.88, and 31.13 ng µl^−1^ in ‘Falcon,’ ‘H-2274,’ and ‘Rio Grande,’ respectively. The highest levels of ABA (0.89 ng µl^−1^), ethylene (0.67 ng µl^−1^), IAA (15.46 ng µl^−1^), and zeatin (49.07 ng µl^−1^) were found in the cultivar ‘Falcon’; the cultivar ‘Rio Grande’ had the higher (GA_3_) and SA with a mean of 1,870, 92.89 ng µl^−1^, respectively. To obtain the detailed separation of the detected phytohormones in this study, multivariate discriminant analysis PCA and HCA were also constructed. Based on the existence of diversity and similarity in the cultivars, we found two major cluster groups, cluster I comprised cv. ‘Falcon,’ and cluster II consisted of the two cultivars (‘H-2274’ and ‘Rio Grande’), which were divided into two subgroups (Figure 7). We further determined by using the scatter biplot method that PC1 (96.5%) and PC2 (3.52%) contents were constituted to be 100% of the total variation. In addition, the relationships between cultivars and phytohormones in tomato seeds and the distribution of phytohormones are given separately based on tomato cultivars (Figure 7). According to the visual classification of the phytohormones, there was a strict correlation among ABA, IAA, ethylene, and zeatin. Such a similar correlation was observed in GA_3_ and SA. However, both two groups showed negative and weak correlations with each other. Our results indicated that ABA, IAA, ethylene, and zeatin were the major phytohormones for the cv. ‘Falcon,’ whereas the cv. ‘Rio Grande’ was rich in SA, and GA_3_ (Figures 7 and 8). Among studied cultivars, the ‘Falcon’ can be differentiated from the ‘Rio Grande’ and ‘H-2274’ depending on the major abundance of phytohormones.

The role of phytohormones under saline conditions is crucial in regulating physiological responses, which will finally lead to adaptation to an unfavourable environment [36]. Based on these facts, previous works have conjectured that endogenous phytohormones were important in response to salt stress [11,37]. For example, ABA has a mediator role in responses to salt stress [12,38,39], and IAA controls almost all aspects of plant life, from seed germination to flowering stages [4]. Amjad et al. [6] reported that the salt-tolerant genotype had a significantly higher amount of ABA and ethylene than the salt-sensitive genotype under saline conditions. A similar result has also been reported by Babu et al. [4] found the amount of IAA and ABA increased as salt treatment increased. Interestingly, in our experiment, it was observed that the most abundant ABA, IAA, ethylene, and zeatin were found in cv. ‘Falcon’ while this cultivar was not resistant to saline conditions considering germination traits under salt stress. These inconsistent results could be explained by the global effect of the different metabolites in the seed of the salt tolerance of the cultivars. Furthermore, the germination percentage and germination speed are affected by the amount of GA_3_ and ethylene in the seed, especially in saline conditions. That is to say, the germination percentage of the seed is decreased when the seed has low GA_3_ content [27].

## 4 Conclusions

The selection of salt-tolerant cultivars could be a wise solution to minimize the harmful effects of salinity. Biochemical variability offers a valuable tool for investigating salt tolerance mechanisms in tomatoes, and it will be appreciated to find high-tolerant tomato cultivar(s) to saline conditions. Screening of the complex correlation of biochemical content is the most critical requirement to fill the gap in knowledge about the relationships between biochemical profile and seed germination characteristics. In the study, multivariate discriminant analyses were constructed and demonstrated that tomato cultivars were separated from each other by the amino acid, organic acid and phytohormone contents. Considering germination traits of tomato seeds, cv. ‘H-2274’ was more tolerant to salinity than others, depending on high proline and citric acid assays. Based on our results, the biochemical parameters of the seed species could be used for assuming tolerance to saline conditions, although it is not clear that tolerance is in critical stages of plant growth.
